# Application of the Caprini risk assessment model for deep vein thrombosis among patients undergoing laparoscopic surgery for colorectal cancer

**DOI:** 10.1097/MD.0000000000024479

**Published:** 2021-01-29

**Authors:** Xiuying Lu, Weirong Zeng, Lin Zhu, Lu Liu, Fengmei Du, Qing Yang

**Affiliations:** a Department of Operating Room; b Department of Gastrointestinal Surgical Oncology, Sichuan Cancer Hospital & Institute, Sichuan Cancer Center, School of Medicine, University of Electronic Science and Technology of China, Chengdu, China.

**Keywords:** Caprini risk assessment model, colorectal cancer, deep vein thrombosis, laparoscopic surgery

## Abstract

Application of the Caprini risk assessment model was explored in patients with deep vein thrombosis (DVT) after laparoscopic colorectal cancer surgery.

This study was a prospective study. The risk factors for DVT were assessed with a survey at baseline and on the morning of surgery, first day after surgery and sixth day by using repeated blood vessels on color Doppler ultrasound of the lower limbs, and the intraoperative and postoperative conditions were recorded.

Among 148 surgical patients, 24.3% had asymptomatic DVT. According to the risk stratification, the incidence of DVT was related to the Caprini score (*P* < .001). The area under the curve of the Caprini model was 0.701 ± 0.047 (95% CI: 0.609–0.793, *P*<.001). The Youden index was 0.368, while the critical point was 10.5 in the Caprini model, corresponding to a sensitivity of 0.806 and a specificity of 0.563. Age, cardiovascular disease, intraoperative blood loss, postoperative fever, preoperative preparation, and hospital stay were higher in DVT patients than in patients without DVT. Moreover, the incidence of DVT in patients with a lithotomy position was higher than that in patients with a scissors position. In binary logistic regression analysis, the independent risk factors for DVT development were age, intraoperative blood loss, and preoperative preparation time.

The Caprini model can be used for the prediction of venous thromboembolism in laparoscopic colorectal cancer surgery patients. The thrombosis risk assessment model must be established in line with patients undergoing endoscopic malignant tumor surgery.

## 1 Introduction

Deep vein thrombosis (DVT) is a venous reflux disorder caused by abnormal condensation in deep veins that often occurs in the lower extremities. Venous thromboembolism (VTE), including DVT and pulmonary embolism (PE), is the third most common vascular disease after cerebral stroke and cardiovascular disease.^[1]^ DVT is one of the common complications during the perioperative period of surgery.^[2]^ DVT can influence the postoperative rehabilitation of patients and can even result in fatal PE.^[3]^ The incidence of DVT in postoperative colorectal cancer patients is up to 37% to 46% when the corresponding prevention interventions are not taken during the perioperative period in surgical in-patients.^[4,5]^ The advantages of laparoscopic surgery are minimal trauma, minimal pain, a fast recovery, and a short ambulation time.^[6]^ However, according to some other reports, lower limb blood flow can be affected by other conditions, such as increases in abdominal pressure by CO_2_ pneumoperitoneum in laparoscopic surgery and an intraoperative Trendelenburg position. The incidence of postoperative DVT is also elevated by various factors, such as blood hypercoagulability states caused by changes in coagulation and fibrinolysis during laparoscopic surgery and injury to venous blood vessels.^[7,8]^ Hence, early risk assessment and targeted prevention measures are key for reducing the incidence of DVT. According to the VTE prevention guide made by the American College of Chest Physicians 2012, it is clear that all patients need to undergo VTE risk assessment, and preventive treatment should be performed for high-risk patients.^[9]^

In recent years, people have been devoted to analyzing thrombosis risk assessment models to take preventive measures for high-risk patients. Cabrini RAM is the most widely used and fully verified tool for risk prediction.^[10]^ Nevertheless, Caprini studies are mostly retrospective studies. Recently, the inaccuracy of evaluations has been caused by using standard preventive measures in high-risk patients in most hospitals.^[11,12]^ Therefore, it is necessary that prospective studies of this model are used in laparoscopic colorectal cancer surgery. This study aims to verify the predictive value of the Caprini risk assessment model in the formation of DVT during the perioperative period in laparoscopic colorectal cancer surgery patients, providing a reference for choosing the appropriate assessment tools in clinical work.

## 2 Materials and methods

### 2.1 Research design

A prospective cohort study was used, and patients who underwent laparoscopic resection for rectal cancer surgery were continuously involved from September 23, 2019 to October 1, 2020. DVT was screened by using color Doppler ultrasound of blood vessels in the lower limbs during the perioperative period in all patients from Sichuan Cancer Hospital in China, and then the Caprini risk assessment model was used to mark each surgical patient. The actual incidence and affecting factors of DVT at different DVT risk levels were evaluated. The study was approved by the Ethics Committee of Sichuan Cancer Hospital (approval number: SCCHEC-02–2019-007), and patients and families signed informed consent forms.

### 2.2 Inclusion and exclusion criteria

Inclusion criteria:

(1) colorectal malignancy was confirmed by pathology;
(2) patients who received surgical treatment;
(3) patients without embolism or a history of anticoagulation;
(4) patients without severe complications (including cardiopulmonary dysfunction, renal failure, multiple organ failure, incision infection, intraoperative/postoperative hemorrhage, anastomotic fistula, etc); and
(5) informed consent.

Exclusion criteria:

(1) patients with positive DVT feedback by preoperative color Doppler ultrasound examination;
(2) patients who needed a second surgery;
(3) patients who were converted to open operations during laparoscopic surgery; and
(4) patients who quit the clinical trial.

### 2.3 Research tools

The research tools used in this study included the following.

(1) A general homemade form: including name, age, body mass index, diagnosis, history of drinking and smoking, central venous catheterization, complications, pathological results, etc.
(2) Clinical data (form for intraoperative and postoperative conditions): including intraoperative bleeding, surgery duration, surgical position, blood transfusion, DVT prevention, complications, hospitalization time, and so on.
(3) Caprini risk assessment model (Table 1): the Caprini risk assessment model was established based on clinical trials and previous studies and was used for the risk assessment of DVT in-patients.^[13]^ Along with the substantial research on diseases and clinical risk factors, the Caprini risk assessment tool was constantly upgraded to relatively mature in 2010. Approximately 40 risk factors, including age, surgery, and clinical laboratory testing, were ranked from 1 to 5 points by risk level. The scores were obtained if any factor was detected. Otherwise, there was no corresponding score. The total score of each patient was collected. Caprini was incorporated in the 9th guidelines by the American College of Chest Physicians, suitable for non-orthopedic surgery patients. Different DVT-relevant preventive measures are recommended for different risk stratifications.^[9]^ In reference to the research of Lobastov, the risk of DVT was stratified into low-risk (0–1), moderate (2), high-risk (3–4), high-risk (5–8), high-risk (9–11), and high-risk (≥12) subgroups.^[14]^
(4) DVT diagnostic criteria: according to the Chinese Cancer Related to VTE Prevention and Treatment Guideline (2019 edition), color Doppler ultrasound was used as the preferred imaging examination for the presumptive diagnosis of DVT.^[15]^ An ultrasound Scanner 1202 color Doppler ultrasonic diagnostic scanner was used in this study. It has a line matrix ultrasound probe, which has a frequency between 5 and 10 MHz. The sampling value was in the central blood vessels, within the incidence angle of the sound beam at approximately 60°. The external iliac vein, femoral vein, foretibial vein, and posterior tibial vein were examined by 2-dimensional ultrasound scan. Then, the popliteal vein, fibula veins, gastrocnemius vein, and plantar vein were tested while the patient was in the prone position with 30° of knee flexion. The recently appeared weak echo was found and filled in the veins of both lower limbs. The free end of the thrombus was detected to float with the bloodstream while tracking to the proximal end. Thrombosis was indicated by color doppler flow imaging at an incidence angle of 60° of the sound beam, with no blood flow signals in the lumen and no blood flow signals or incomplete blood-filling signals after adjusting the blood flow velocity scale.

**Table 1:**
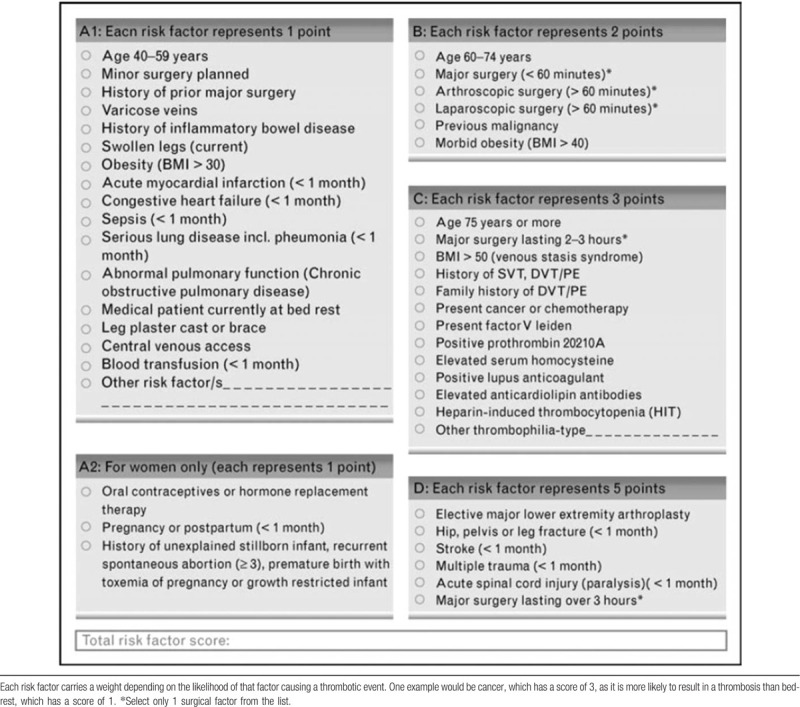
Caprini risk assessement model.

### 2.4 Data collection methods

The patients who underwent laparoscopic resection for rectal cancer in Sichuan Cancer Hospital from September 23, 2019 to October 1, 2020 were visited and informed regarding the surgery as well as purpose and methods of the study by an operating room nurse on preoperative day 1. The patients also had to sign the consent form that day. On the morning of the operation day, the operation room specialized staff consulted the patient's case and collected the general data. Caprini scores were measured and color Doppler ultrasound was performed on the patients, and DVT-positive patients were excluded before surgery. After the operation, the patients returned to the ward and received postoperative care following laparoscopic radical resection for colorectal cancer. Color Doppler ultrasound was used on the first day after surgery and on the sixth day. Then, the results were reported to the primary-care doctors.

To prevent deep vein thrombosis of the lower extremities, preventive measures were taken according to the Chinese Cancer Related to VTE Prevention and Treatment Guideline (2019 edition): when the patient returned to the ward, exercises could be undertaken, such as thrombus exercises; if the condition permitted, the patient was allowed to perform out-of-bed activities on preoperative day 1. With the doctor's advice, an intermittent inflation pressure device was used to compress the lower limbs mechanically under a pneumatic pump pressure of 20 to 30 mm Hg. This treatment lasted for 30 minutes in the antithrombotic mode. It improved the lower limb venous return and reduced venous blood retention. Patients with high-risk DVT (Caprini score≥3 points) were subcutaneously injected with 3075 AxalU nadroparin calcium once a day after postoperative day 2 to 5, based on the risk of bleeding. High-risk patients with postoperative bleeding were prohibited from drug prevention.

### 2.5 Statistical methods

The IBM SPSS 24.0 statistical software package was used for analysis. The data are presented as the $\bar{\chi }$ ±s or M (P25–P75), and the percentage (%) represents the incidence. *T* tests were used to compare the normal distribution of the sample means, nonnormal data comparison was performed by rank tests, and the Chi-squared test was used to test the counting data. Then, the receiver operating characteristic curve was drawn, and the area under the curve (AUC), sensitivity and specificity were calculated. The best diagnostic cutoff point was demonstrated by the largest cutoff point of the Youden index and was used to prevent DVT in the Caprini model. DVT risk was analyzed through multifactor logistic regression, and the difference was statistically significant (*P* < .05).

## 3 Results

### 3.1 Basic information

There were 153 patients who underwent laparoscopic colorectal cancer surgery from September 23, 2019 to October 1, 2020, and 5 patients underwent laparotomy during the intraoperative period. In total, 148 patients participated (81 males, 67 females), the median age was 64 (55–70 years old), the minimum age was 31 years old, and the maximum age was 87 years old. The intermediate body mass index was 22.86 (21.02–24.97), while the minimum was 17.26 and the maximum was 31.25. The operative time ranged from 0.5 to 9.33 hours (median, 3.63 hours (2.92–4.33)). The median intraoperative blood loss was 50 mL (50–100 mL), ranging from 5 to 600 mL. The median length of stay was 14 days (12–17 days), varying from 9 to 28 days. Eighty-five patients undergoing laparoscopic colorectal cancer surgery, 61 patients undergoing laparoscopic colon surgery and 2 patients undergoing laparoscopic colorectal surgery were monitored. All patients underwent mechanical preventive measures after surgery, and 63 patients were under drug prevention. Only 2 participants suffered from urinary tract infections, and no complications occurred, such as surgical site infection, pulmonary infection, or anastomotic leakage.

### 3.2 Prevalence of DVT

No preoperative blood clots were detected by color Doppler ultrasound in any of the patients. However, 36 patients (24.3%) were found to have postoperative DVT on postoperative day 6, including venous thrombosis of the calf muscle (35 patients) and venous thrombus at the popliteal vein branch (1 patient). Four of the DVTs were in both lower limbs, 15 were in the left lower limb, and the others (17 patients) were in the right lower limb. The patients were treated after being reported to doctors. During the follow-up period, no patients with symptomatic DVT or DVT complications were observed.

### 3.3 Applications of the Caprini model

**Figure 1:**
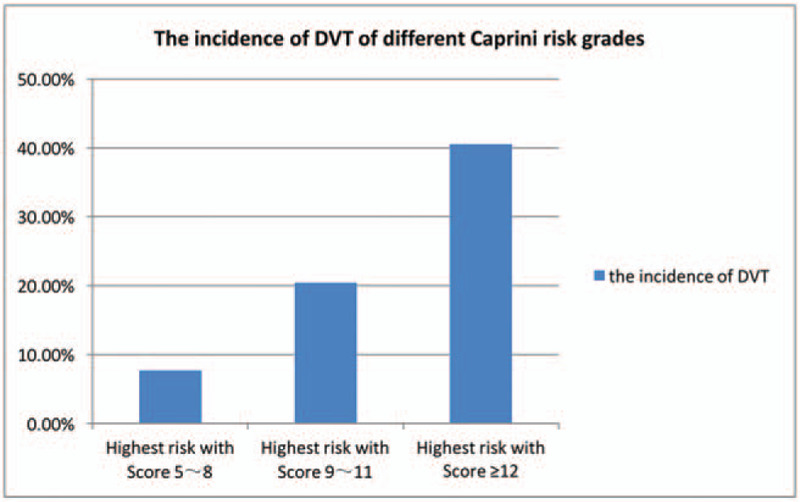
The incidence of deep vein thrombosis of different Caprini risk grades.

The range of Caprini scores was from 7 to 15, with a median of 11 points (9–12). The Caprini scores of all patients were higher than 5 points, the high-risk (5–8) subgroup accounted for 7.69%, the high-risk (9–11) subgroup accounted for 20.41%, and the high-risk (≥12) subgroup accounted for 40.54%. The differences were statistically significant (*P* = .018) (Table 2, Fig. 1).

**Table 2:** Comparison among deep vein thrombosis risk, Caprini scores and risk levels. (n = 148).

Risk level	DVT (—)	DVT (+)	Z	*P*
Caprini RMA score	10 (9–11)	11 (11–12)	–3.695	<.001
Highest risk with Score 5–8	12 (92.31)	1 (7.69)	Z = 8.001	.018
Highest risk with Score 9–11	78 (79.59)	20 (20.41)		
Highest risk with Score ≥12	22 (59.46)	15 (40.54)		

DVT = deep vein thrombosis.

**Figure 2:**
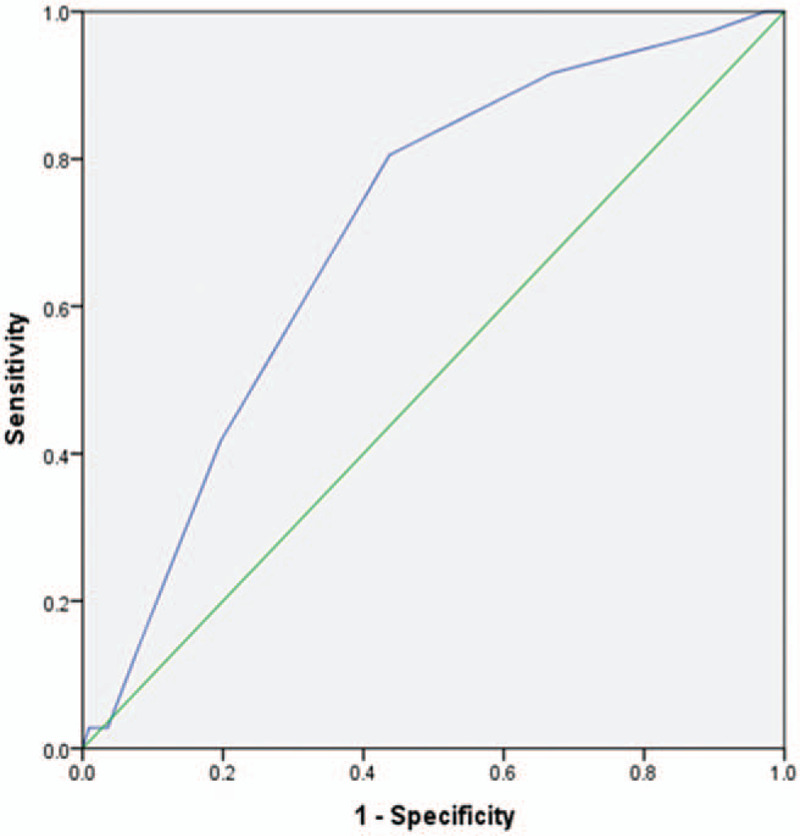
Receiver operating characteristic curve for Caprini scores.

In 148 patients undergoing laparoscopic colorectal cancer surgery, the AUC of the Caprini model was 0.701 ± 0.047 (95% CI: 0.609–0.793, *P<*.001) (Fig. 2). The critical point was 10.5 with a Youden index of 0.368, which corresponded to a sensitivity of 0.806 and a specificity of 0.563.

### 3.4 Single factor analysis of DVT risk

In these patients, according to single analysis, age, cardiovascular disease, intraoperative surgical posture, intraoperative blood loss, postoperative fever, preoperative preparation timem, and hospitalization time could vary the incidence of DVT (*P* < .05) (Table 3).

**Table 3:** Single-factor analysis of deep vein thrombosis incidence risk.

	Thrombus occurrence n (%)			
Subjects	No	Yes	Statistics	*P* value
Gender				
Male	61 (75.3)	20 (24.7)	χ^2^ = 0.013	.909
Female	51 (76.1)	16 (23.9)		
Age				
<45	8 (88.9)	1 (11.1)	Z = –4.479	<.001
45–59	43 (93.5)	3 (6.5)		
60–74	53 (72.6)	20 (27.4)		
≥75	8 (40.0)	12 (60.0)		
Smoking				
No	66 (77.6)	19 (22.4)	χ^2^ = 0.422	.516
Yes	46 (73.0)	17 (27.0)		
Drinking				
No	67 (77.9)	19 (22.1)	χ^2^ = 0.555	.456
Yes	45 (72.6)	17 (27.4)		
CVD				
No	81 (82.7)	17 (17.3)	χ^2^ = 7.672	.006
Yes	31 (62.0)	19 (38.0)		
Respiratory aliments				
No	10 (78.5)	28 (21.5)	χ^2^ = 3.348	.067
Yes	10 (55.6)	8 (44.4)		
Diabetes				
No	10 (77.7)	29 (22.3)	χ^2^ = 1.547	.214
Yes	11 (61.1)	7 (38.9)		
Hypertension				
No	86 (77.5)	25 (22.5)	χ^2^ = 0.783	.376
Yes	26 (70.3)	11 (29.7)		
Intraoperative position				
Scissors	60 (84.5)	11 (15.5)	χ^2^ = 5.782	.016
Lithotomy	52 (67.5)	25 (32.5)		
Operation time				
<3	34 (79.1)	9 (20.9)	Z = –0.693	.806
3–3.99	41 (77.4)	12 (22.6)		
4–4.99	21 (70.0)	9 (30.0)		
≥5	16 (72.7)	6 (27.3)		
Intraoperative blood loss				
<150	100 (78.7)	27 (21.3)	χ^2^ = 4.566	.033
≥150	12 (57.1)	9 (42.9)		
Intraoperative transfusion				
No	107 (77.0)	32 (23.0)	χ^2^ = 1.104	.293
Yes	5 (55.6)	4 (44.4)		
Fever after operation				
Without	103 (79.8)	26 (20.2)	χ^2^ = 9.489	.002
With	9 (47.4)	10 (52.6)		
Chemotherapy/radiotherapy				
No	75 (76.5)	23 (23.5)	χ^2^ = 0.115	.734
Yes	37 (74.0)	13 (26.0)		
Preoperative preparation	5 (4–7)	7 (5.25–10)	Z = –4.193	<.001
Length of stay	14 (12–16)	16 (14–19.75)	Z = –3.330	.001

### 3.5 Multi-Factor analysis of DVT risk

The incidence of DVT was treated as a dependent variable, while the independent variables were indexes with statistical significance, in single-factor analysis (age, cardiovascular disease, intraoperative surgical posture, intraoperative blood loss, postoperative fever, preoperative preparation time, and hospitalization time). The results of binary classification logistic regression analysis showed that variations in age, intraoperative blood loss, and preoperative preparation time were the main risk factors influencing the incidence of DVT (*P* < .05) (Table 4).

**Table 4:** Logistic regression analysis of deep vein thrombosis risk factors.

					95%CI	
Risk factor	B	SE	*P*	OR	Lower	Upper
Constant	−7.699	1.475	.000	0.000		
Age	1.116	0.347	.001	3.209	1.627	6.330
Intraoperative bloos loss	1.421	0.597	.017	4.142	1.286	13.340
Preoperative preparation time	0.233	0.084	.006	1.262	1.070	1.489

## 4 Discussion

In this study of over 148 hospitalized medical patients, we found that differences in age, intraoperative blood loss, and preoperative preparation time were risk factors for DVT.

The study showed that the high-risk DVT patients were mainly laparoscopic colorectal cancer surgery patients, with an incidence of 24.3%, which was consistent with the results of Lobastov. In this study, the risk of VTE in patients with malignant tumors was more than 4 to 7 times higher than that in patients without malignant tumors.^[16,17]^ In patients who developed VTE, cancer and other factors are among the strongest covariates associated with VTE.^[18]^ The high coagulation state is enhanced by thrombin from malignant tumors, which promotes tumor adhesion.^[19,20]^ The usage of CO_2_ pneumoperitoneum in laparoscopic surgery results in blood viscosity and solidification. In addition, the risk of DVT is increased in the venous blood stasis state, which is caused by a higher intra-abdominal pressure than the venous blood backflow pressure in the lower limbs, resulting in obvious expansion of the femoral vein and reduction of blood flow.^[8]^ The dorsal elevated position, convenient for operation practices, is normally chosen in laparoscopic surgery. Lower limb blood reflux is blocked in this position.^[7]^ During the postoperative period, a high blood coagulation state, leading to postoperative DVT formation, is facilitated by less activity in a short time, imbalances in acids and alkali, and electrolyte stimulation by intestinal paralysis.^[21]^ The operating time is longer with this surgery compared with open surgery due to some highly difficult manipulations, such as lymph node cleaning under a peritoneoscope. Therefore, patients are increasingly stimulated, causing vascular endothelial cell injury. For laparoscopic colorectal cancer surgery patients, the prevention of perioperative VTE needs to be achieved to avoid the occurrence of venous thrombosis.

PiranS et al showed that advanced age is a high risk factor for DVT.^[22]^ With increasing age, body metabolism continuously slows down, and vein elasticity and venous blood velocity are significantly lowered. In addition, the blood components and blood flow dynamics vary, consistent with the degradation of organ functions in elderly individuals. A high blood condensation state develops with local blood vessel hardening, inducing the risk of thrombosis.^[23,24]^It has been demonstrated that age is the main high-risk index of DVT incidence in laparoscopic colorectal cancer patients, and the incidence of DVT was 60% once the age was above 75 years old. Overall, the senile patients were the main concern in this study.

The increasing incidence is closely related to venous thrombosis in the condition of intraoperative blood loss. Thus, it is statistically significant that intraoperative blood loss is correlated with postoperative venous thrombosis (*P* = .03).^[25,26]^ The study showed that intraoperative blood loss was a high-risk factor for DVT incidence. The occurrence of DVT was 42.9%, while the amount of intraoperative blood loss was greater 150 mL.^[27]^ Laparoscopic colorectal cancer surgery has a large scope of operation, leading to damage to the blood vessel wall and exploration of intravascular subcutaneous collagen. This surgery readily induces the activation of platelet and blood coagulation factors. The compensatory mechanism is activated to maintain environmental balance in the body, along with blood loss augmentation. The balanced environment of blood is destroyed with compensatory hyperplasia of red cells and other factors, causing hemoconcentration. Coagulation and stasis occur with hemoconcentration, leading to venous thrombosis.^[25]^ Patients who suffer from large intraoperative blood loss accompanied by unstable hemodynamics should be treated with hemostatic fluid infusion, transfusion or other anti-shock therapies. However, anticoagulant drugs cannot be used in the early stage, which causes a high coagulation state by the accumulation of clotting substances in the body. Finally, the risk of DVT is aggrandizing.^[22]^

The preoperative preparation time refers to the days from patient admission to the hospital for surgery. Preoperative hospitalization lessens the activity level of patients. In an early statement, the incidence of VTE was found to increase with activity reduction.^[28]^ In this study, it was proven that preoperative preparation time was a factor that induced DVT in patients who underwent laparoscopic colorectal cancer surgery. Patients easily had DVT if they had a preoperative preparation time of 7 days or more.^[29]^ In a study of hip fracture patients, the hospital stays of patients with DVT were longer than those of patients without DVT.^[30]^ In Zhang's study, delays in surgical time were a risk factor for DVT during the preoperative and postoperative periods in hip fracture patients.^[31]^ Shibuya found that the extension of hospital stay was another risk factor for DVT and pulmonary embolism.^[32]^ Patients can barely reach the peak period of psychological barriers during the preoperative surgery period.^[33]^ The longer the preparation time of patients is, the stronger they suffer from helplessness. The variation in psychological function is affected by patients’ mental states. Consequently, the workflow should be positively improved to shorten the postoperative preparation time, reducing DVT incidence and promoting recovery.

It was easily found in our study that the Caprini model could be used in DVT prediction for laparoscopic colorectal cancer surgery patients. Further research on risk stratification and the assessment of content validity is needed. The AUC of the Caprini model in our study was 0.701 ± 0.047, and the accuracy of the trial could be diagnosed and reflected generally by the area under the receiver operating characteristic curve. It is known that an AUC between 0.5 and 07 indicates a low-efficiency diagnosis, an AUC varying from 0.7 to 0.9 shows moderate efficiency, and an AUC above 0.9 indicates a high-efficiency diagnosis.^[34]^ The test revealed that the Caprini model had moderate efficiency for diagnosing laparoscopic colorectal surgery patients. The result was in accordance with the highest sensitivity and specificity scores of 11 points. The efficacy of Caprini was evaluated in high-risk VTE patients and proceeded with subgroup analysis to predict VTE more correctly. The accuracy was higher when the cutoff value was 11.^[35]^ Once the critical point of the Caprini model was 10.5, the sensitivity was 0.806, the specificity was 0.563, and the Youden index was 0.368. It was shown that the Caprini model has high sensitivity and low specificity in the prediction of DVT in laparoscopic colorectal cancer surgery patients. The Caprini risk level should be further tested.

According to the multifactor analysis in this study, age, intraoperative blood loss, and preoperative preparation time were independent risk factors for DVT in laparoscopic colorectal cancer surgery patients. However, in the Caprini assessment scale, the effects of age were referred, while intraoperative blood loss and preoperative preparation time were not mentioned. The assessment content was not comprehensive for laparoscopic colorectal cancer surgery patients, and further evaluation is needed.

Nevertheless, the study has certain limitations:

(1) only patients with symptomatic thrombosis were included, and they were not monitored by color Doppler ultrasound during the follow-up visit;
(2) it was a single-center study;
(3) during standard prevention, the measurement of medication time was not quintessentially enforced, which might have affected the results.

Further research should be conducted to address the limitations of this study.

## 5 Conclusions

In conclusion, the Caprini model can be used to prevent VTE in laparoscopic colorectal cancer surgery patients. However, the efficacy of the VTE risk level and the evaluation of content during the perioperative period should be further tested, and a thrombosis risk assessment model, applied to patients undergoing endoscopic surgery for malignant tumors, should be explored and established.

## Acknowledgments

We thank Hai Hu and Hui Yang for helpful comments and discussions that improved our work. Two anonymous reviewers helped us further improve the presentation.

## Author contributions

**Conceptualization:** Xiuying Lu.

**Data curation:** Xiuying Lu, Lin Zhu, Lu Liu, Qing Yang.

**Formal analysis:** Xiuying Lu, Qing Yang.

**Funding acquisition:** Xiuying Lu.

**Investigation:** Xiuying Lu, Weirong Zeng, Lin Zhu, Lu Liu, Fengmei Du.

**Methodology:** Xiuying Lu.

**Project administration:** Xiuying Lu.

**Resources:** Xiuying Lu.

**Software:** Lin Zhu, Lu Liu, Qing Yang.

**Supervision:** Xiuying Lu, Qing Yang.

**Validation:** Xiuying Lu, Qing Yang.

**Visualization:** Xiuying Lu, Qing Yang.

**Writing – original draft:** Xiuying Lu.

**Writing – review & editing:** Qing Yang.

## Abbreviations

Abbreviations: AUC = area under the curve, DVT = deep vein thrombosis, VTE = venous thromboembolism.
